# Anaphoric Pronouns and the Computation of Prominence Profiles

**DOI:** 10.1007/s10936-022-09873-9

**Published:** 2022-04-16

**Authors:** Barbara Tomaszewicz-Özakın, Petra B. Schumacher

**Affiliations:** Department of German Language and Literature I, Linguistics, University of Cologne, Albertus-Magnus-Platz, 50923 Cologne, Germany

**Keywords:** Prominence, Reference, Pronoun, Demonstrative, Comprehension

## Abstract

Previous research has investigated anaphoric resolution at the anaphor. Using a self-paced reading study we show that prominence profiles, i.e. the ranking of the referential candidates for anaphoric resolution, are dynamically established as discourse unfolds. We compared four types of context sentences introducing two referents and found that the cost of the computation of the prominence profile depends on the alignment of prominence-lending features, namely ‘left edge’, ‘agent’, ‘subject’. Cost occurs as referents become available. Further downstream, we contrasted two types of pronouns in German, personal pronoun vs. demonstrative pronoun. By the time the pronoun is encountered, profile computation is already complete, as indicated by the lack of interaction between context and pronoun type. An effect of pronoun reveals that resolution is driven by the form-dependent strength with which an interpretation is obtained (demonstrative pronouns being more stable than personal pronouns). The results also indicate that two prominence-lending features – subjecthood and agentivity – compete with each other.

## Introduction

Research on the comprehension of pronouns has shown that multiple features contribute to the resolution of different types of anaphoric expressions, among others agentivity, topicality, subjecthood and coherence relations (cf. e.g., Arnold, 2010). These features impact production and interpretation probabilities in subtle ways (Rohde & Kehler, 2014). This raises the question how these features are weighted against each other during the ranking of the referential candidates for anaphoric resolution, i.e. what the respective prominence profiles are. Our study has two goals. While previous research has mainly focused on the processing correlates of referential resolution at the anaphoric expression, we investigate the establishment of prominence profiles while the mental model is built up. This in turn will allow us to better understand the nature of the processing costs for anaphor resolution. In the following, we first introduce the notion of prominence and its relevance for discourse representation before providing an overview of the features contributing to referential resolution. We then present a self-paced reading study that examined the reading times for four different contexts (rendering different prominence profiles and thus specific rankings of the referential candidates) and two types of pronouns (personal pronouns and d(emonstrative)-pronouns).

### Prominence in Discourse

Communicative content involves a massive amount of information and in order to be digestible and retrievable, it is structured by speakers and hearers in a particular way. We argue that the notion of prominence as it has been proposed for grammar (Himmelmann & Primus, 2015) plays a key role in organizing information in discourse representation. While the notion of prominence has been used quite loosely in the literature (and often interchangeably with terms like salience, accessibility, activation, familiarity or centering), Himmelmann and Primus (2015) develop a new framework in which prominence represents a common organizing principle that is applicable to all levels of linguistic description. In particular, they postulate three criteria for prominence in grammar: (i) linguistic units of equal type (e.g., syllables, co-arguments of a predicate) compete for the status of being in the center of attention; (ii) the status of linguistic units may shift dynamically; (iii) prominent units act as structural attractors in their linguistic domain. Von Heusinger and Schumacher (2019) illustrate how these criteria apply to the level of discourse, where individual referents, time points, eventualities and propositions serve as basic linguistic units that need to be organized in discourse representation. They argue that prominence in discourse can be characterized as (i) relational, (ii) dynamic, and (iii) attracting linguistic operations, and they further demonstrate that other notions like salience, accessibility, activation or centering can be derived from this more general account of prominence.

Let’s briefly consider the three definitional criteria. (i) Prominence is a relational property that singles out one element from a set of elements of equal type, i.e. in our case it singles out a particular referent from the set of currently relevant referents and establishes an ordered set of referents.Following from this there is one entity that is the center of attention (cf. Grosz et al., 1995) or that is most strongly activated or accessible (e.g., Chafe, 1976; Ariel, 1990; Gundel et al., 1993). The ranking of referential candidates relies on so-called prominence-lending cues such as grammatical function, thematic role, topicality or position (see below). (ii) The prominence of an entity can shift as the discourse unfolds, so that a particular referent may be prominent at point t_1_ but another referent may be promoted to represent the most prominent entity at point t_2_. These changes in the prominence profile are mediated by prominence-lending features associated with referential expressions and result in the updating of the discourse representation structure. This is for instance reflected in topic discontinuity (Givón, 1983) or perspectival shifts (Hinterwimmer, 2019). Dynamicity has generally been assumed by dynamic discourse representation theories (Kamp & Reyle, 1993; Lascarides & Asher, 2007) but it has played no or only a minor role in reference resolution research, where accessibility or activation is typically viewed as static (e.g., the Givenness Hierarchy by Gundel et al., 1993 postulates a strict correspondence between referential form and cognitive status). (iii) Prominent units are structural attractors, i.e. they serve as anchors for the larger structures they are constituents of. Accordingly, they may license more operations than their competitors. For example, prominent units license perspectival operations (Hinterwimmer, 2019) or serve as temporal anchors for entire propositions (Becker & Egetenmeyer, 2018); they dispose of an increased remention capacity (cf. Givón, 1983) and allow for more variation of referential expressions (see the implicational relation of cognitive statuses in Gundel et al., 1993).

This more precise notion of prominence in discourse is capable of replacing alternative accounts. In particular, it highlights the relational nature of prominence and dismisses prominence as an inherent property of a particular form (contra static notions). Furthermore, there is hardly any consideration of the other functional contributions – dynamicity and structural attraction – in alternative accounts. Hence basic assumptions of referential activation (Chafe, 1976; Lambrecht, 1994) and familiarity (Prince, 1981) can be reconstructed on the basis of the relational criterion of prominence. Likewise, conjectures about centers (Grosz et al., 1995) are reflected in the competitive nature underlying the relational notion of prominence. And while Centering Theory considers dynamicity, transitions only apply between two utterances and not within the larger discourse segment; yet a fully fledged theory of prominence should also take into account prominence-lending cues from paragraphs and larger texts, such as discourse topicality, perspective taking or narrative structure. Salience is a notion inspired by the cognitive sciences where it marks an entity that stands out. In semantic theory, it indicates a ranked set of entities (Lewis, 1979; von Heusinger, 1997), which again can be derived from the relational notion of prominence. Salience theories further acknowledge the dynamic aspect of prominence via context change functions (see also Chiarcos, 2011). The entailment relations assumed in accessibility theories (Ariel, 1990; Gundel et al., 1993) can be captured by structural attraction inherent in our notion of prominence. We thus use the term prominence in the following as a more explicit characterization of the organization of entities in discourse representation and their ability to shape the upcoming discourse.

Our focus in the current paper is on the relational nature of prominence and the question when it is established. The ranking of referents in discourse has consequences for anaphoric resolution in that the use of different types of referential expressions correlates with the degree of prominence of a particular discourse entity (as elaborated on by accounts of accessibility, e.g., Ariel, 1990; Gundel et al., 1993). Personal pronouns in English or zero pronouns in Italian are claimed to preferably select the most prominent entity as their referent, demonstrative pronouns usually exclude the most prominent entity as a potential referent, and definite noun phrases typically refer to a less prominent entity. This has also been discussed in the context of referent accessibility in the literature (see Arnold, 2010) but the notion of prominence we are adopting here is not confined to referent selection but represents a more general organizing principle (including the notion of a dynamic change of discourse representation as well as structural attraction).

To illustrate how prominence profiles are established take for instance the following example in which two referents are introduced in (1a), *Laura* and *Kate.* These two entities are represented in discourse in such a way that one is characterized to be more prominent than the other. Potential prominence-lending features are grammatical function, thematic role and position. *Laura* being the subject, the agent and the first mentioned referent outranks *Kate* in all of these properties, hence *Laura* is considered more prominent. As a consequence, the personal pronoun in (1b) is preferably interpreted as referring to *Laura*. When a speaker wants to refer to *Kate*, she typically uses a more marked form – such as a stressed pronoun (1c) or a demonstrative pronoun in German (1d) – to indicate that she is not referring to the most prominent entity in the current discourse.

The interpretive preferences can therefore be derived from the prominence profiles of the involved referents. Crucially, reference resolution in discourse is a matter of preferences, which are violable. In particular, personal pronouns are potentially ambiguous but preferences should emerge as soon as a solid prominence profile is available. Other referential forms (e.g., demonstrative pronouns) are more restricted in their choice of referent but also show a certain degree of freedom with regard to reference selection.

### Prominence Ranking of Referents

Pronouns have very little descriptive content except for some basic features (number, person, gender). They are anaphoric, i.e. they inherit their reference from other referring entities. When processing a pronoun, the mental representation of a discourse entity is accessed from the mental model, and the choice of the referent is guided by the prominence ranking. Pronouns are claimed to select a high ranked referent. Or put differently, the accessibility of a referent is dependent on its ranking with respect to the other entities activated in the current discourse, and the choice of anaphoric expression to refer to an entity depends on the rank of that entity.

As observed above, personal pronouns tend to pick up the most prominent, most easily accessible entity. In addition to the semantics of the predicate and inter-clausal coherence relations, several other factors may contribute to the prominence status of a referent: its syntactic function (subject > object > other), its thematic role (agent > patient), its information status (topic > non-topic), as well as its position within the clause (first mention > second mention, or alternatively recency: second mention > first mention) (see Arnold, 2010 for an overview). Subject preference in pronoun resolution, as well as order of mention, the parallel function heuristics and the contribution of coherence relations have been well documented (e.g., Crawley et al., 1990; Clark & Sengul, 1979; Gordon et al., 1993; Chambers & Smyth, 1998; Järvikivi et al., 2005a, b; Koornneef & van Berkum, 2006; Kaiser & Trueswell, 2008; Rohde & Kehler, 2014; inter alia). In contrast to ‘regular’ personal pronouns, the stressed versions, as in (1c), have been shown to signal the anaphoric link with the lower ranked referent (cf. Balogh, 2003 for experimental evidence).

Kehler and colleagues (2008) furthermore propose to distinguish between preferences that guide next mention biases and those that influence choices in production. Accordingly, the processing of personal pronouns is influenced by distinct probabilistic expectations of the hearer regarding (i) which entity will be mentioned next and (ii) which referential expression will be used to mention this entity. Using sentence completion tasks with a pronoun prompt and a free prompt condition, it is argued that next mention biases are guided by coherence relations and that the production bias (i.e. pronominalization) is influenced by grammatical function and topicality (Kehler & Rohde, 2019; Kehler et al., 2008).

Different forms of referential expressions track referents in distinct positions in the prominence ranking (Ariel, 1990; Gundel et al., 1993). The prominence hierarchy fits in with a general property of communication: the tendency to keep utterances as informative as necessary, but no more informative than necessary (Grice’s, 1975*Maxim of Quantity*). Thus, shorter and less specific referential expressions such as pronouns are preferred for the entities that are already prominent in the current context. In some languages, however, there are pronouns with a more specialized referential function. In German d-pronouns have been claimed to have a strong bias against being resolved to referents that are the subject or topic of the immediately preceding sentence (Bosch et al., 2007; Hinterwimmer, 2015)^1^:

The fact that the functions of different types of pronouns enable the tracking of different prominence status of referents in the current discourse makes them a perfect diagnostic tool for establishing the prominence profile of referential candidates in a given utterance. Numerous grammatical features have been discussed that lend prominence to an entity. Here we focus on the interplay of grammatical function, sentential position and thematic role. The contribution of grammatical function to the resolution of personal and demonstrative pronouns has been investigated in various languages and, all other things being equal, it has been shown that personal pronouns prefer subject antecedents whereas demonstrative pronouns prefer object antecedents (e.g., for German: Bosch et al., 2003; Finnish: Kaiser & Trueswell, 2008; Dutch: Kaiser, 2011). However, it has also been observed that a single prominence-lending cue does not suffice to account for resolution preferences and that different referential forms can be sensitive to different prominence-lending features (e.g., Brown-Schmidt et al., 2005; Kaiser & Trueswell, 2008).

As far as positional information is concerned, edge placement has been discussed as a candidate for high prominence, i.e. entities that are processed initially and lastly are privileged over entities in medial position (Himmelmann & Primus, 2015). In particular the left edge or initial position has been identified as standing out. This may be due to the incremental nature of processing and the desire to place important information initially, or it might have an information structural basis, since sentence-initial entities often represent topics, contrastive elements or frame setters. Furthermore, if edges carry a particular highlighting function, then initiality or finality do not just represent a prominence-lending feature, but these positions serve as structural attractors (i.e. are privileged positions). In previous research, personal pronouns have registered a tendency to refer to initial arguments (but with a lot of flexibility) and demonstrative pronouns have been resolved more robustly in favor of final arguments (e.g., for German: Bosch et al., 2007; Wilson, 2009; Finnish: Kaiser & Trueswell, 2008). To confront the fact that nominative case and positional prominence often coincide, Ellert (2011) tested context sentences with comparative constructions that have two nominative arguments in German (e.g., “the doctor is friendlier than the cook”). Following the canonical order, the personal pronoun resolved towards the first mentioned referent and the d-pronoun towards the second mentioned referent.

Thematic role information also impacts the ranking of referential candidates. We follow the proposal of generalized semantic roles or proto-roles, according to which arguments exhibit one or more of the following features, rendering them more agent- or patient-like (Dowty, 1991; Primus, 1999). According to Dowty’s proposal, proto-agents are characterized by volitional involvement, sentience (and/or perception), causation and autonomous motion and subsume the roles of agent, experiencer, causer, possessor. Proto-patients undergo change of state, are causally and volitionally affected, and stationary (relative to other participants), including patient, stimulus or possessed as more specific roles. While the contribution of thematic roles has been investigated for personal pronouns in English (e.g., Arnold, 2001; Kehler et al., 2008; Stevenson et al., 1994), their impact on the resolution of personal vs. demonstrative pronouns has only been studied for German (Schumacher et al., 2016, 2017): using active accusative verbs and dative experiencer verbs, personal pronouns showed a preference for the proto-agent and d-pronouns for the proto-patient.

Let’s consider the interplay of grammatical function, thematic role and position. In the first clause of (2) (as well as in (3a) discussed below) the cues to the prominence ranking of the two referents are perfectly aligned: the subject is the agent and it is in sentence-initial position, while the object is the patient and the last mentioned entity. Do these prominence-lending properties play an equal role in contributing to the prominence ranking?

In languages with a less rigid word order than in English, it is possible to disentangle the individual contribution of the different prominence cues. In the German example in (3a’), with the non-canonical object-before-subject word order, the object/patient is in the initial position, while the subject/agent is mentioned second. Thus, in (3a’), the cues are partially aligned: subjecthood and agentivity are aligned, but they are separated from initial position. Crucially, German also allows for the separation of subjecthood and agentivity in the case of verbs that assign dative case to the highest thematic roles, (4) (as a shorthand, let us call them ‘dative verbs’ and those in (3) ‘accusative verbs’). For dative verbs, the experiencer object qua sentience satisfies one of the proto-agent entailments. In the canonical argument order (4a) the dative object whose thematic role is experiencer surfaces initially, thus (proto-)agentivity and initial position are aligned. In (4a’) subjecthood and initial position coincide. We will discuss these alignment patterns in detail in the next section.

In a series of experiments Schumacher and colleagues (2016, 2017) established that agentivity is a stronger cue for pronoun resolution during comprehension than subjecthood in German. With accusative verbs, (3), the personal pronoun *er* was preferably interpreted as referring to the nominative agent, and the d-pronoun as referring to the accusative patient. These preferences were independent of the word order manipulation, but in the non-canonical order the preferences for *er* were weaker. With dative verbs, the word order did influence the interpretive biases. The canonical order (4a) elicited an interpretive bias for the object experiencer (the proto-agent) for the personal pronoun and for the lower thematic role for the d-pronoun. The non-canonical condition (4a’) registered no interpretive preferences. Both findings can be explained by full vs. partial alignment of prominence cues in the context sentence.

These interpretive preferences have been found in offline and online tasks (Schumacher et al., 2016, 2017). The question that remains is whether prominence profiles are computed at the pronoun (i.e., when a referentially deficient expression is encountered) or as the discourse unfolds (i.e., already during the processing of the context sentence). In this paper we thus investigate the establishment of the prominence ranking between the two referents from the context sentences.

## The Current Study

The experiment, a self-paced reading task, was designed to elaborate on a specific aspect of the findings inSchumacher et al. (2016, 2017): the processing of the prominence profile associated with the two referents introduced in the context sentence. In those prior studies, it was determined that multiple cues contribute to prominence in German, such that agentivity is a stronger cue for prominence than subjecthood, yet agentivity alone cannot do the job. Those results were obtained by comparing the anaphoric preferences for the German personal pronoun *er* and the d-pronoun *der* in the contexts of accusative and dative verbs, as discussed and illustrated above in (3) and (4). The different combinations of canonical/non-canonical word order and the two types of verbs in the context sentence resulted in different combinations of three prominence-lending cues with their respective rankings exemplified in (5).

- (5) a. grammatical function: subject > object
- b. thematic role: proto-agent > proto-patient (cf. Dowty, 1991; Primus, 1999)
- c. position: sentence-initial > non-initial

This renders the feature alignments of the highest members in the individual rankings as presented in Table 1. The first referent introduced in the canonical sentences with accusative verbs, (3a), enjoys the status of a subject and of an agent and occurs sentence-initially, which means that in this case the prominence cues under investigation are fully aligned: referent 1 outranks referent 2. In the non-canonical word order, (3a’), referent 2 outranks referent 1 because it has two features of high prominence, while referent 1 has only one.

**Table 1:** Distribution of prominence-lending features for the two referents

	word order	features of the 1^st^ referent	features of the 2^nd^ referent
accusative verbs	canonical (3a)	initial + subject + agent	–
	non-canonical (3a’)	initial	subject + agent
dative verbs	canonical (4a)	initial + agent	subject
	non-canonical (4a’)	initial + subject	agent

With dative verbs, subjecthood is separated from agentivity. In both canonical and non-canonical word orders, referent 1 carries two high prominence features, while referent 2 has only one of them. They differ in that referent 1 in the canonical word order holds the agent property, and in the non-canonical word order referent 1 holds the subject property.

Processing differences for these structures at the pronoun (Schumacher et al., 2015, 2016, 2017, as summarized in “Prominence ranking of referents”) suggest that prominence profiles are a reflection of the interaction of prominence-lending cues. However the point at which prominence profiles are established – i.e., when discourse unfolds or when a referentially deficient entity must be resolved – needs further scrutiny. Our predictions for i) the dynamic establishment of the prominence profiles associated with the four types of context sentences and ii) pronoun resolution are presented in the next section.

## Predictions

### Predictions for the Context Sentence

We posit that prominence profiles are dynamically constructed as a discourse unfolds. An utterance like (3)/(4) that contains two referents requires the establishment of a ranking of the referents because only one entity should ideally stand out and be in the center of attention (see also Grosz et al., 1995). This is important for predictive forward processing, i.e. the calculation of a referent’s role in the developing discourse, and may impact both next-mention biases and the choice of referential forms (cf. e.g., Kehler et al., 2008). Accordingly, we expect to observe processing consequences from the computation of prominence profiles as the referents in the context sentence become available, in particular at the sentence-final main verb, when the interpretation of the whole event is established. Thus while linguistic input is processed incrementally and certain prominence-lending cues become immediately available at the arguments, other cues only become available at the predicate. Verb-specific information is particularly essential to determine prominence relations since agentivity has been identified as a key cue for referential processing (Schumacher et al., 2016, 2017). Verb information is crucial for the identification of thematic role and grammatical function in German, as illustrated by distinct mappings of thematic role, grammatical function and case by verb types – e.g., *besuchen* (‘visit’ – nominative subject agent and accusative object patient), *danken* (‘thank’—nominative subject agent and dative object patient), *interessieren* (‘interest’ – accusative object experiencer and nominative subject stimulus), or *imponieren* (‘impress’ – dative object experiencer and nominative subject stimulus) – or by subject-verb agreement in double nominative constructions (see Ellert, 2011). Hence while some prominence-lending cues already become available at the arguments allowing for a preliminary ranking, the complete featural setup emerges only at the predicate.^2^

Based on the alignment of the prominence-lending features sketched in Table 1, we predict a cline with increasing costs for decreasing feature alignment at the verb where all cues are available. If feature alignment is a numerical matter, then the canonical accusative context (with three features aligned) should have an advantage over the other contexts (with two alignments each). If, on the other hand, the first position is privileged, the prominence profile of the canonical accusative context (3 features aligned in the first position) should be easier to compute than the non-canonical accusative context (only 1 feature in the first position) and no difference should accrue between the two dative contexts (2 features each).

In the sentence processing literature an alternative account exists that dispenses with discourse-based prominence and that only considers sentence level argument processing.^3^ Indeed an immense body of research has investigated argument ordering in German. This research has typically contrasted case ambiguous argument alternations and measured processing costs at a disambiguating predicate, which yielded longer reading times and specific electrophysiological profiles for non-canonical argument orders (e.g., Bader & Meng, 1999; Bornkessel & Schlesewsky, 2006). Crucially, however, when the first argument unambiguously carried case cues (as in our study), no effect of argument linearization emerged at the first or the second noun phrase in accusative verb contexts (Frisch et al., 2002 – when determiner and noun were presented separately, a canonicity effect emerged on the determiner; see Rösler et al., 1998; Matzke et al., 2002). Note further that no results were reported for the predicate position in the Frisch et al. study. In research on dative verbs, dative arguments were processed just as nominative arguments in initial position (i.e. no processing disadvantage) and dative experiencer verbs (like the ones used in our study) engendered thematic reanalysis effects at the dative experiencer predicate (irrespective of argument order) relative to a predicate with canonical nominative-before-dative order (Bornkessel et al., 2003). Given that the arguments in our stimuli are unambiguously case marked, this indicates that no effects of argument order are expected based on a discourse-independent argument processing approach.

#### Predictions for the Pronoun

Previous self-paced reading studies investigating pronoun resolution found differences as a function of contextual factors, e.g., longer reading times for inconsistent (contextually unexpected) personal pronouns utilizing implicit causality verbs (Koornneef & van Berkum, 2006) or coherence relations (Chambers & Smyth, 1998; Wolf et al., 2004). Similarly, Hinterwimmer and Brocher (2018) investigated binding constellations and reported longer reading times for structurally illicit pronominal reference. In contrast, the present study does not examine mismatching (less preferred) pronouns but rather the online resolution of personal and d-pronouns in licit contexts. At the position of the pronoun, we expect to observe a difference between the two pronoun types, possibly interacting with the variation of prominence-lending cues in the context. Accordingly, the two types of pronouns serve as a diagnostic for our question whether prominence profiles are established online or only when required by a pronoun. We thus predict in line with our hypothesis for the context sentence, that pronouns should have immediate access to the previously established prominence profiles. This may then be dependent on the robustness of the profiles (reflecting feature alignment and/or weighting following the context manipulations below).

As far as the influence of the different prominence profiles is concerned, event-related brain potential (ERP) data revealed an effect of pronoun type, which disappeared following the non-canonical accusative verb contexts (Schumacher et al., 2015). This suggests that resolution was encumbered when the first argument carried only the positional feature. In contrast, visual world and offline data indicated that interpretive preferences diminished following non-canonical dative verb contexts (Schumacher et al., 2016, 2017). This points toward a competition between the subject cue and the thematic role cue during interpretation. In our self-paced reading experiment, at the position of the pronoun, we should find an interaction between pronoun type and context type, if the prominence profiles are computed at the pronoun, i.e. when a referentially deficient entity must be resolved. If, on the other hand, prominence profiles are dynamically computed as the discourse unfolds and this computation is already complete by the time of the processing of the pronoun, the effect of pronoun type should be independent of the effect of context. Furthermore, in the ERP study the d-pronoun engendered processing costs compared to the personal pronoun (Schumacher et al., 2015). But the d-pronoun also yielded more reliable interpretive preferences, while the personal pronoun elicited more variability in visual world eye tracking and offline referent identification tasks (Schumacher et al. 2016, 2017). This yields mixed predictions for reading times. Costs of the d-pronoun could arise either because the exclusion of the most prominent candidate from the set of referents is resource consuming or because the d-pronoun represents an unexpected continuation (i.e., default referential maintenance predicts a personal pronoun). In turn, the cost of the personal pronoun may arise due to its ambiguity (mirrored by more interpretive variability in previous work, e.g. Bosch et al., 2007, Wilson, 2009, Ellert, 2011 contra Bouma & Hopp, 2007).

### Participants

Twenty-five native speakers of German (13 women, all between 18–30 years of age) participated in this study after giving written informed consent. All of them had normal or corrected-to-normal visual acuity and were not diagnosed with a reading disability by their own report.

### Materials and Design

We tested the two sets of verbs in two sub-experiments in a single study. The main motivation for combining the two experiments in a single study was that, given the differences in the semantics of the verbs, we could not include the four different types of prominence cue alignments (see Table 1) in the context as four levels of the same condition.

In both sub-experiments we crossed two levels of the variable Pronoun Type (*er*, *der*) and two levels of Context Type (canonical, non-canonical). Sample items for each of the four conditions in Experiment 1 and 2 are shown below, (6)-(7). The “|” symbol indicates the division into regions, which represent the segments for the self-paced reading as well as the regions for analysis. We included a ‘distractor sentence’ between the context sentence and the target sentence, in order to avoid any slowdown in the context sentence to spill over onto the pronoun, which was always at the beginning of the target sentence following the connective *aber* (‘but’). The same connective *aber* was used in all of the items and fillers; the adversative connector signals contrast or the violation of an expectation generated by previous discourse and it may enhance resolution towards an experiencer argument (see Schumacher et al. 2016 for effects of the adversative connector).

In each sub-experiment 40 items were constructed for each of the 4 conditions (for a total of 160 items). The 320 test items from both sub-experiments were distributed across 4 lists. Each list also contained 28 fillers with gender disambiguated referents and thus unambiguous pronouns in the target sentences; half of them contained passive context sentences.^4^ The order of the items and fillers was pseudo-randomized such that no more than two sentences of the same type could follow one another. One third of all the sentences, including all of the fillers, was followed by a comprehension question.

### Procedure

The self-paced reading experiment was run on a desktop computer using the OpenSesame software (Mathôt et al., 2012). In a moving-window paradigm, the participants first saw the full sentence with all the words masked by dashes. At the press of the spacebar each consecutive phrase was revealed (consisting either of a single word, a determiner phrase or a prepositional phrase) while the previous phrase became masked again. One third of the stimuli was proceeded by a comprehension question requiring a ‘yes’/’no’-response. The experiment took about 30 minutes to complete.

### Data Analysis

Overall the accuracy on the comprehension questions was 83%, and we kept the data from all participants. In the reading time data, we removed all outlier reaction times (RTs) below 200 ms and above 4000 ms (0.08% of the experimental and filler data). For each subject we computed residual reading times (Ferreira & Clifton, 1986; Trueswell & Tanenhaus, 1994), which were used for statistical analyses without further trimming. Residual reading times account for the differences in: (a) word length^5^ (because there is no linear relationship between the number of characters in a word and the required reading time), (b) word position in a sentence (because it also has no linear effect on reading times), (c) the log-transformed trial number (because readers speed up as the experiment progresses), and (d) individual differences in participants' reading speeds. Such a two step-model of reading time data analysis was used in Jaeger et al. (2008), Hofmeister (2011), and Hofmeister et al. (2013) (for the implementation in R see hlplab.wordpress.com/2008/01/23/modeling-self-paced-reading-data-effects-of-word-length-word-position-spill-over-etc./). Crucially, residualization corrects for the individual differences by taking the entire data set into account, both items and fillers, whereas including trial as a random effect in the mixed-effects regression model would only take into account experimental trials ignoring fillers. As the second step, we fitted a mixed-effects regression model of residualized reading times in R version 3.4.1 (R Core Team, 2017) using the lme4 package version 1.1–13 (Bates et al., 2015). Predictor variables included Context Type (canonical, non-canonical) and Pronoun Type (*er*, *der*) and an interaction. We used ANOVA-style sum-coded contrasts to test for main effects in the presence of an interaction. We included random intercepts and slopes for participants and items whenever supported by the data (Barr et al., 2013). In cases of convergence failures or of zero/perfectly correlated random terms with very low standard deviations, the random effects structure was simplified following Baayen et al. (2008). The *p*-values were obtained using the Satterthwaite approximation implemented in the lmerTest package (Kuznetsova et al., 2017).

### Results

#### Experiment 1 Accusative Verbs 

**Fig. 1:**
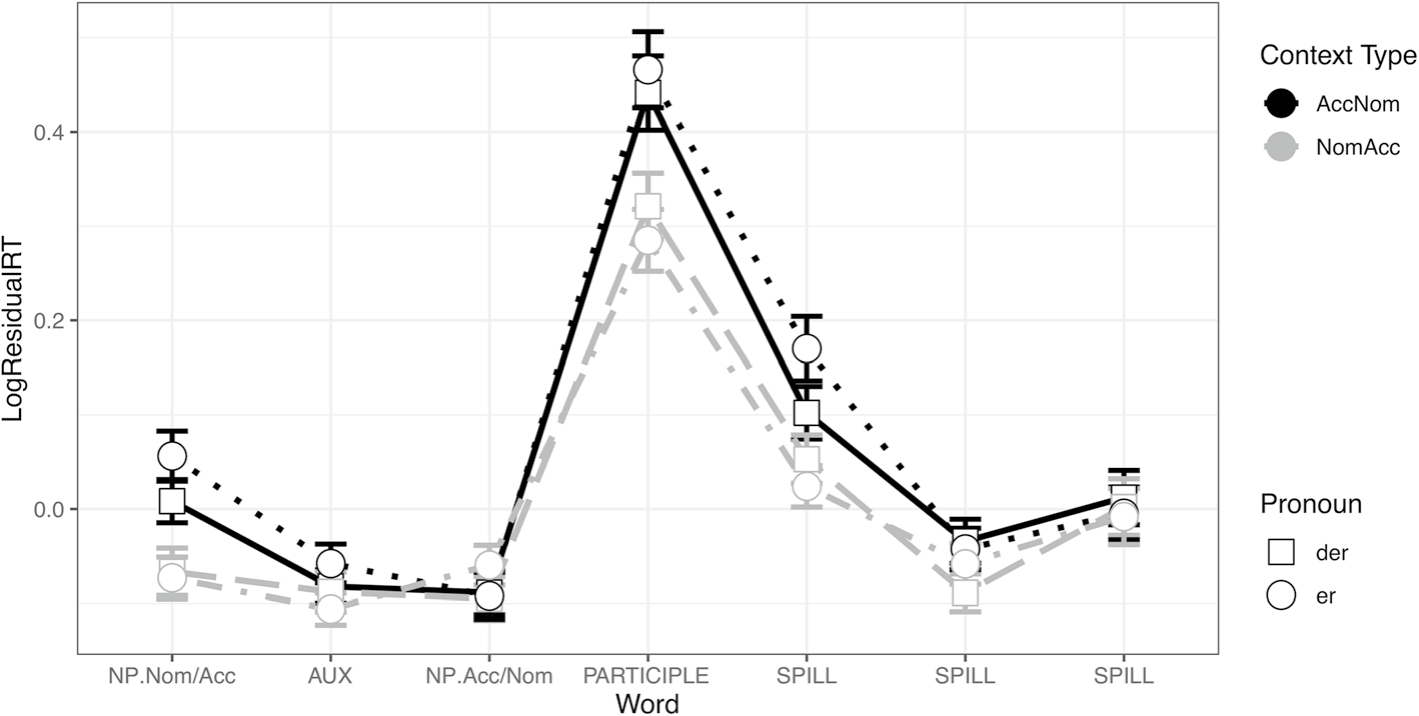
Residual reading times at the context sentence in Experiment 1 (Accusative Verbs)

In the context sentence in Experiment 1 there were significant differences in reading times in region 1 (NP._Nom/Acc_) and in regions 4 (participle), 5 and 6 in the spillover, as seen in the plot in Fig. 1. In region 1 (NP._Nom/Acc_),^6^ accusative objects were read slower than nominative subjects (β = 0.103, SE = 0.022, t = 4.638, *p* =  < 0.0001). In region 4 (participle),^7^ the verbal participle was read slower in the non-canonical order condition (β = 0.153, SE = 0.033, t = 4.633, *p* =  < 0.0001). This effect persisted in region 5,^8^ the first segment of the distractor sentence (β = 0.097, SE = 0.028, t = 3.442, *p* = 0.001) and in region 6^9^ (β = 0.037, SE = 0.018, t = 1.991, *p* = 0.047). As expected, there was no effect of Pronoun Type in any of the regions, and no significant interactions, since the pronoun has not been processed yet.

**Fig. 2:**
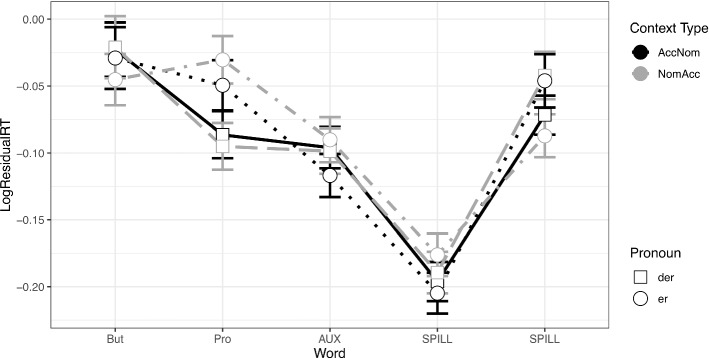
Residual reading times at the target sentence in Experiment 1 (Accusative Verbs)

In the target sentence, we found no differences in reading times on the conjunct ‘aber’ (see the plot in Fig. 2). In the following region containing the subject pronoun,^10^ there was a significant difference in the reading times between the two types of pronouns: d-pronouns were read significantly faster (β = 0.051, SE = 0.017, *t* = 2.924, *p* = 0.004). There was no effect of Context Type (β = -0.005, SE = 0.021, *t* = -0.251, *p* = 0.8) and no significant interaction (β = -0.027, SE = 0.036, t = -0.74, *p* = 0.46). There were no differences in RTs in the subsequent regions. Model estimates for regions 1 through 11 are all included in the Appendix.

#### Experiment 2 Dative Experiencer Verbs

**Fig. 3:**
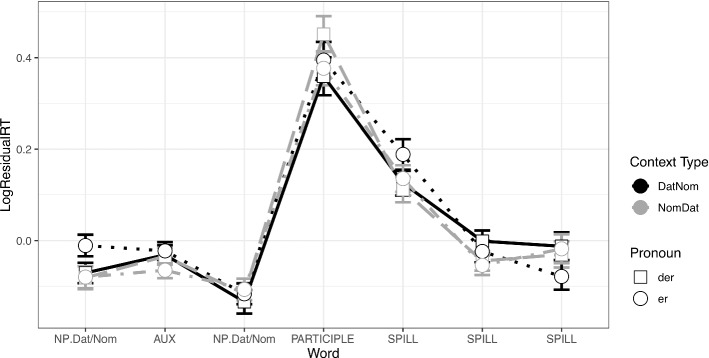
Residual reading times at the context sentence in Experiment 2 (Dative Experiencer Verbs)

In the context sentence with the dative experiencer constructions we observed no significant differences in reading times in any of the regions (Fig. 3). (Some of the differences approached significance; model estimates for regions 1–11 are included in the Appendix.)

**Fig. 4:**
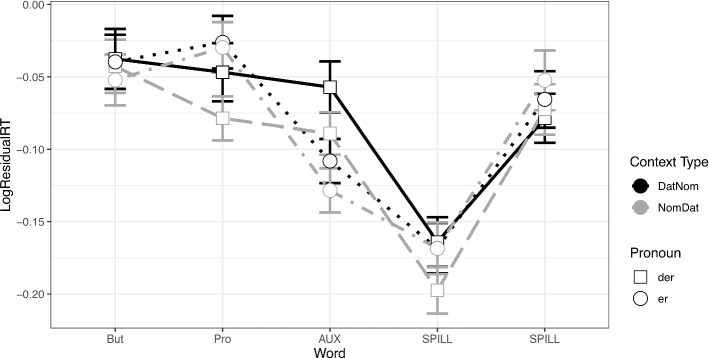
Residual reading times at the target sentence in Experiment 2 (Dative Experiencer Verbs)

In the target sentence, there were no differences in RTs at the connective *aber* (‘but’). At the region of the subject pronoun,^11^ a marginal effect of Pronoun Type emerged (β = 0.036, SE = 0.021, *t* = 1.719, *p* = 0.09), with faster reading times for the d-pronouns. Subsequently, at the auxiliary,^12^ the RTs for d-pronouns became significantly slower (β = -0.046, SE = 0.017, *t* = -2.69, *p* = 0.011) compared to the personal pronoun. There was also a marginal effect of Context Type in this region (β = -0.026, SE = 0.015, *t* = -1.693, *p* = 0.09). There were no significant interactions. At region 11 (adverb), there were no significant differences. See Fig. 4 .

## Discussion

This study investigated the time-course of the potential establishment of prominence profiles by comparing the incremental processing of four different types of context sentences and revealed longer reading times at the first argument and the sentence-final predicate for the non-canonical accusative constructions and no differences between the two dative verb contexts. The study further examined the processing of personal and d-pronouns and found faster reading times immediately at the d-pronoun for all contexts followed by increased reading times in the spill-over region of d-pronouns for the dative experiencer contexts. The context and pronoun effects are discussed separately in the following sections.

### Prominence Profile of the Context Sentence

The results for the context sentences support our predictions that prominence profiles are dynamically constructed as a discourse unfolds and that processing cost increases as feature alignment decreases. The incremental nature of the self-paced reading task revealed how the ranking of the two referents according to their prominence takes place during sentence processing. Importantly, we observed two separate patterns for the two word orders with accusative verbs. The partial alignment profile (accusative-before-nominative context) was more costly to process than the full alignment profile (nominative-before-accusative context). No processing differences were observed between the two word orders with dative verbs.

In the non-canonical contexts with accusative verbs (in Experiment 1), there were increased reading times for the sentence-initial accusative object compared to the nominative subject in the same position. This is expected given that the parser treats the canonical subject-before-object order as the default; but remember that previous research on sentence processing reported no electrophysiological differences between nominative- and accusative-initial arguments (Frisch et al., 2002). This suggests that task differences (reading sentences vs. reading small texts; grammaticality judgments vs. comprehension questions) may impact argument processing, such that text comprehension triggers a deeper processing. The increased reading times at the initial accusative argument may either indicate that the parser has sufficient information to expect a transitive structure and cost accrues due to building a transitive – rather than an intransitive – structure (cf. primacy of minimal structure; Bornkessel & Schlesewsky, 2006) or that the absence of further prominence-lending cues exerts demands during prominence computation. The former explanation can be discarded in light of the absence of processing costs during sentence reading (Frisch et al., 2002). We thus argue that the accusative argument does not represent a good candidate for an initial argument and this incurs processing costs. Subsequently, the parser quickly recovered from this initial surprisal, and the following two regions, the auxiliary and the nominative subject, were read no slower than those in the canonical word order condition. At the final segment of the sentence, the participle, there was again an increase in the reading times for the non-canonical condition, and it persisted into the following two regions in the distractor sentence. This supports our prediction that the computation of prominence profiles should affect processing as the referents in the context sentence and their roles in the event become available (that is at the sentence-final main verb, when the interpretation of the event is completed). The observed effect indicates that at the end of the sentence, when its propositional content is established, both the truth-conditions and the pragmatics are processed – i.e., the ranking of the two referents and their associated prominence-lending cues is processed at the level of propositional content. A conceivable alternative would involve a slowdown for non-canonical argument order at the sentence-initial object as well as the immediately following segments. Instead, we found no difficulties at the syntactic level, but rather a cost for the special pragmatic content of the sentence, i.e. the prominence profile.

A limitation of our experimental design is that it presented the context sentences out of the blue. As a consequence, the accusative-initial sentences were rather marked and not information structurally licensed. Previous research indicates that proper licensing of non-canonical structures can facilitate processing (e.g., Hörnig et al., 2006; Schumacher & Hung, 2012; Weskott et al., 2011 for German non-canonical structures in contexts). If richer contexts are constructed in future research, we predict that the disadvantage of the initial accusative argument might disappear once the object is properly licensed by context. In this case, another prominence-lending cue must be added to the prominence profile (like topicality, contrast or givenness) and it would have to be determined how grammatical function, thematic role and the corresponding information structural cue interact with each other during the establishment of the prominence ranking.

The fact that Experiment 1, with accusative verbs, revealed the nature of the cost of the computation of the prominence profile with this particular methodology is important for our second finding – that with dative verbs, in Experiment 2, the two word orders do not result in differences in processing. We attribute this to equal cost of prominence ranking. In Experiment 1, the profile with 3 prominence-lending features aligned at the left edge was easier to process than the partial alignment profile with 1 feature at the left edge. In Experiment 2, in the canonical order, left edge placement and proto-agentivity coincide (2 features); in the non-canonical order, left edge placement and subjecthood coincide (2 features). Experiment 2 further provides us with direct evidence that the first segments, the dative object or nominative subject, are processed alike, in contrast to accusative objects in Experiment 1. German allows for unmarked nominative- and dative-initial structures, which is reflected in the absence of reading time differences at the initial argument in Experiment 2. But accusative-initial structures signal a marked structure, hence the longer reading times observed in Experiment 1.

The difference between the two experiments indicates that feature alignment is not just a matter of the number of accumulated features, but rather that the left edge is privileged: the prominence profile of the canonical accusative context (3 features aligned in first position) is easier to compute than the non-canonical accusative context (only 1 feature in first position) but the two dative contexts (2 features each) are no different.

The dative verb contexts in Experiment 2 provided further evidence for the approach to prominence ranking in terms of multiple weighted constraints (Arnold et al., 2000; Badecker & Straub, 2002; Järvikivi et al., 2005a, b, 2014; Kaiser & Trueswell, 2008) or discrete probabilistic paths (Kehler et al., 2008). In this experiment, in both canonical and non-canonical contexts the prominence-lending cues were misaligned: subjecthood was separated from agentivity (recall Table 1). In the canonical context, the higher ranked referent was a sentence-initial (proto-)agent; in the non-canonical context, it was a sentence-initial subject. We found that during incremental processing, both rankings received similar prominence profiles. These results are consistent with two kinds of theoretical approaches to dative experiencer constructions in German. One approach takes the dative-nominative order of arguments to be the canonical one in German, while the nominative-dative order is facilitated by subject-before-verb being the default argument order in the language (Haider, 1993). According to the other approach, neither of the argument orders is more canonical/marked than the other, precisely because the first argument is either a subject or an agent and both of these features have similar consequences for the syntax (Primus, 1999; Barðdal et al., 2014) . Our result that agentivity and subjecthood are on equal footing in the processing of the prominence profiles underscores the cross-linguistic variability of heuristics such as ‘subject preference’. Indeed, the research on thematic roles has shown that thematic information guides incremental argument processing independently of structural information (Bornkessel & Schlesewsky, 2006; Ferreira, 2003). For prominence profiles, both subjecthood and agentivity impact the referential ranking. Concerning pronoun resolution, future research needs to investigate whether subjecthood and agentivity impact next mention biases and production biases in distinct ways (cf. Kehler & Rohde, 2019).

Overall, we find evidence that prominence profiles are established during text comprehension. The incremental construction of prominence profiles is particularly evident by the fact that at the pronoun no effect of context emerged. The findings indicate that prominence profiles are dynamically constructed and made available as discourse unfolds. They demonstrate that prominence profiles are not computed when needed by a pronoun but instead are established incrementally. This way prominence profiles can be used to generate predictions for the resolution of upcoming pronouns.

Prominence profiling is further based on feature alignment rather than accumulation (see longer reading times for non-canonical accusative contexts despite accumulation of two features on second argument). The first position thus bears a privileged role during the computation of referential prominence. This also confirms the claim that the left edge is a privileged position and serves as a structural attractor (cf. Himmelmann & Primus, 2015). As far as prominence-lending cues are concerned, the pronounced effect on the verb supports the claim that thematic role information contributes a central cue to prominence calculation, since this information is supplied by the predicate. It further fosters the idea that prominence is relational because it is at this point when the different referents can be weighed against each other.

### Processing Pronouns

Based on prior findings we predicted that pronouns should have immediate access to the previously established prominence profiles. We hypothesized that if prominence profiles are dynamically computed in the unfolding discourse, this computation is already complete by the time of the processing of the pronoun and there would be no interaction between the effect of context and pronoun type at the position of the pronoun. However, we also expected that feature alignment, differing between the two experiments, would have some effect on pronoun resolution. Indeed, we found differences between the two experiments indicating that feature alignment in the prominence profile plays a role. In the accusative experiment, Experiment 1, we found that d-pronouns were read significantly faster than personal pronouns, irrespective of the canonicity of the preceding context, which fits in with their more specialized semantics: d-pronouns typically select for antecedents that are not the highest in the ranking, while personal pronouns can select either. Accordingly, with accusative verbs the prominence profiles of the context sentences allowed for easy identification of the less prominent referent, which was reflected in faster reading times for d-pronouns. The slower reading times for personal pronouns can be attributed to their referential ambiguity. In terms of prominence, this could also be modelled as a function of the strength with which an interpretation is obtained (see below on code prominence).

In Experiment 2, with dative verbs, d-pronouns also behaved in accordance with their more specialized function. D-pronouns were read faster than personal pronouns, but the reading times at the following region, the auxiliary verb, were significantly slower following d-pronouns than personal pronouns. This spillover effect suggests that the actual resolution of a d-pronoun is a slow, costly process when there is no clear ranking of the referents (irrespective of the position, the dative object is the more agentive argument, while the nominative argument is the patient). This has already been indicated in prior studies (Schumacher et al., 2015, 2017). A d-pronoun requires the identification of a lower ranked referent, but in both contexts in the dative verb experiment this identification was not as easy as in the accusative verbs experiment. In the accusative verbs experiment, in the canonical sentence context, the lower ranked referent was an entity lacking any prominence-lending features and, in the non-canonical context, it had just one prominence feature (initial position). In the dative verb experiment, in both contexts, the lower ranked referents bore the prominence features agent or subject; just the absence of the position feature placed them lower in the ranking. The agent and subject features clashed with the interpretive bias of the d-pronouns incurring the processing cost that we observed. This further suggests that an anti-topic bias in and of itself (as proposed by e.g., Bosch et al. 2003 and Hinterwimmer, 2015 for initial arguments) is not sufficient to account for the resolution preferences of d-pronouns. An anti-topic bias on its own cannot explain the differential behavior of d-pronouns in Experiments 1 and 2.

Let’s now consider the question whether d-pronouns are simply more specialized than personal pronouns, or whether personal pronouns are actually underspecified/ambiguous as suggested above. As noted in “Prominence ranking of referents”, in languages like English anaphoric pronouns pick as their reference the most prominent referent in the antecedent sentence. However, other languages have a larger inventory of pronominal expressions. For example, Spanish has null subject pronouns, which exhibit a subject bias, as well as overt pronouns, which have no interpretive biases. In (8a) the null pronoun is preferentially interpreted as referring to the subject, while the overt pronoun in (8b) was equally likely matched with the subject and the object (Alonso-Ovalle et al., 2002; Filiaci, 2010; Filiaci et al., 2014).

Similarly, in Italian the null pronoun also has a subject antecedent bias, but the overt pronoun, on the other hand, clearly biases objects (as tested on Italian materials parallel to (8a-b) in Carminati, 2002, Filiaci, 2010; Filiaci et al., 2014). Thus, the overt subject pronoun in Italian has a bias towards the less prominent referent, but its counterpart in Spanish is ambiguous.

Our Experiments 1 and 2 indicated that the German personal pronoun *er* in the subject position is ambiguous, as compared to the d-pronoun *der*. It has long been established that *der* is more specialized in its referential choice than *er* (Bosch et al., 2003; Bosch & Umbach, 2007; Hinterwimmer, 2014; Schumacher et al., 2016). *Der* has a strong bias, i.e. it selects for an entity that is not the most prominent one. Several prior studies found this effect with *der*, however, the evidence for interpretive biases with *er* has been somewhat mixed. For instance, Bosch et al. (2007) found only a weak preference for subjects in both canonical and non-canonical conditions. More tellingly, a visual world eye-tracking study by Wilson (2009) revealed that in the canonical context *er* had an initial preference for the object, which later switched to a preference for the subject. In the non-canonical context, however, no preferences at all were observed. The fact that subjecthood cannot be the main factor guiding the resolution of *er* is also indicated by Ellert’s (2011) visual world study. Here the two antecedent referents were provided in a comparative construction (canonical ‘The doctor is friendlier than the cook.’; non-canonical ‘Friendlier than the cook is the doctor.’) and thus both referents were in the nominative case. In the canonical context *er* and *der* diverged in their preferences: *er* selected the first mentioned referent, *der* the second mentioned, but this effect emerged late. In the non-canonical context, however, both pronouns selected for the second mentioned referent already at an early point in time. The online studies on which we based our materials, Schumacher et al. (2015, 2017), also warranted a conclusion that *er* is indeed more neutral than *der* and we replicated their results. Rather than being in complementary distribution with *der*, *er* is pragmatically unmarked in comparison to *der* and hence more flexible in the choice of its antecedent (at least in simple contexts like the ones typically employed in the research on referential interpretation).

The pragmatic source of this difference between *er* and *der* can be evidenced by their divergent behavior in quantified contexts. Consider first how in the English examples in (9), the pronoun *her* can find its referent in the antecedent ‘a wife’ in (9a), yet in (9b) despite the pragmatic salience of a set of wives the same pronoun cannot find a referent (Büring, 2011).

- (9) a. Every man who has a wife should bring her along.
- b. *Every married man should bring her along.

In parallel examples in German, however, only the d-pronoun cannot find a referent (Patel-Grosz & Grosz, 2017), while the personal pronoun appears to be pragmatically licensed. These judgments have been experimentally tested by Martin (2017), who found that in German, in sentences like (10), personal pronouns were generally accepted (but d-pronouns never were), whereas their counterparts in English showed much larger variability.

Our results clearly support the view that *der* imposes specific pragmatic requirements on its context and that *er* is more pragmatically neutral.

This difference further allows us to observe that the prominence profiles established by the contexts have implications for the processing of the pronouns. Being more constrained with respect to its referential choices, the d-pronoun must exclude the most prominent entity as a potential referential candidate. The personal pronoun, being less constrained, is satisfied with any referent and can opt for a sufficiently good interpretation. This has consequences for the use of the different prominence profiles. The observation that the d-pronoun following dative verb contexts evoked reading time increases in post-pronoun regions indicates that rejecting one of the two potential referents in this context is taxing (while this processing step is not required following the accusative verb contexts). The respective prominence profiles seem to be weaker, yielding a certain degree of uncertainty with respect to the ranking of the referents. This further points to a competition between subjecthood and agentivity during pronoun resolution.

In contrast to Experiment 1, in Experiment 2 we found that the misalignments in prominence cues did interfere with the interpretive biases of the pronouns. In Experiment 1 the d-pronouns, which have stronger interpretive preferences, were easily processed in both non-canonical and canonical context conditions, because the features subject and agent were aligned. In Experiment 2, on the other hand, d-pronouns were difficult to process in both context conditions as reflected in the longer RTs on the segment immediately following the pronoun. In Experiment 2 subjecthood and agentivity were separated and this misalignment significantly disrupted the identification of the antecedent for the d-pronoun. Thus, like in the online experiments of Schumacher et al. (2015, 2017), we obtained evidence that the processor is sensitive to multiple cues during pronoun resolution, however, prominence profiles are established online and not only when required by a pronoun. Whether this competition between prominence-lending cues is due to cue interaction or specific functions of the two cues during predictive processing (cf. the Bayesian approaches; e.g., Kehler & Rohde, 2019) must be addressed in future research.

Overall, the findings from the pronoun and its spill-over region indicate first and foremost a difference between the two referential forms. We have argued that demonstrative pronouns are more specialized and more stable in the choice of their referent in contrast to personal pronouns. The data thus suggest that prominence is not just a matter of discourse prominence (i.e. the profile made available by the context sentence), but also of the referential form: in order to refer to entities at different levels of the prominence hierarchy, the language system has discrete forms at its disposal, which signal the prominence status of their respective referent. The strength with which a form gives rise to a particular interpretation (its code prominence) is thus reflected in the reading times at the pronoun.

Finally, the processing patterns at the pronouns observed here during self-paced reading diverge in certain respects from other online measures obtained by visual world eye tracking and the recording of ERPs during reading using the same experimental design. This suggests that the different methodologies are sensitive to distinct aspects underlying pronoun processing. The major difference is that the current data show an effect of referential ambiguity for the personal pronoun – but note that this could also be inferred from the less robust interpretive preferences in visual world eye tracking (Schumacher et al., 2017). The ERP data evoked a biphasic processing pattern for the d-pronoun over the personal pronoun, which has been attributed to expectation-based processing and discourse updating respectively (Schumacher et al., 2015). Expectation-based processing is based on the prominence profile and may feed into both the expectation for a particular referential form and the interpretation preferences. Differences due to the context manipulation across methods may thus reflect different sensitivities of the methods to these two sources of expectation. And finally, discourse updating has been associated with the particular function of d-pronouns to signal a topic shift (Weinrich, 1993), i.e., ‘warn’ the addressee that the prominence profile of the discourse may change. A method with a high temporal resolution like ERP is more apt to pick up distinct functional processes during reading.

## Conclusion

The current self-paced reading study has shown that prominence profiles are established as a discourse unfolds. We argue that the differences in reading times observed during the first sentence are due to the construction of a ranked set of referents. Crucially, the ranking is not established post-hoc at the pronoun but is used immediately for forward processing. At the pronoun, we further observed an effect of the strength with which an interpretation is obtained (demonstrative pronouns being more stable than personal pronouns) and additional costs for the d-pronoun following less clear prominence profiles in the dative verb contexts (which indicates that two prominence-lending features – subjecthood and agentivity – compete with each other).

## Figures and Tables

**Fig. 5:**
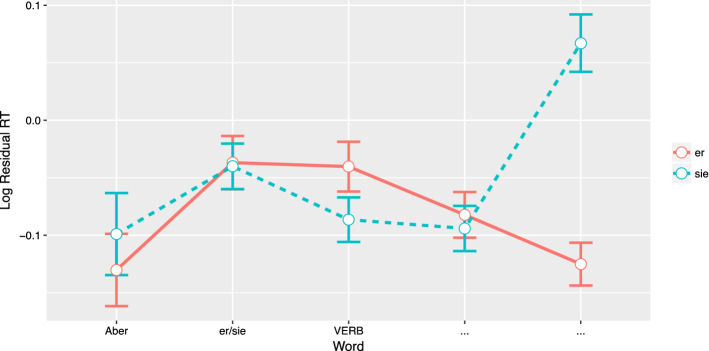
RTs in the Fillers (not matched after the auxiliary verb**)**

## Appendix

See Tables 2, 3 and Fig. 5.

**Table 2:** Estimates for mixed effects model of residual reading times in Experiment 1 Accusative Verbs

	Fixed effects					
		Estimate	Std. Error	t value	Pr( >|t|)	
Region 1 *Nominative Subject / Accusative Object*	(Intercept)	− 0.018	0.034	− 0.525	0.6	
	ContextType	0.103	0.022	4.638	< .0001	***
	PronounType	0.019	0.022	0.865	0.39	
	ContextType:PronounType	0.052	0.044	1.182	0.24	
Region 2 *Auxiliary*	(Intercept)	− 0.083	0.023	− 3.686	< .001	***
	ContextType	0.028	0.018	1.578	0.11	
	PronounType	0.003	0.018	0.142	0.89	
	ContextType:PronounType	0.042	0.036	1.182	0.24	
Region 3 *Accusatiive Object / Nominative Subject*	(Intercept)	− 0.084	0.028	− 2.988	0.006	**
	ContextType	− 0.013	0.022	− 0.609	0.54	
	PronounType	0.016	0.022	0.722	0.47	
	ContextType:PronounType	− 0.039	0.044	− 0.889	0.37	
Region 4 *Participle*	(Intercept)	0.379	0.058	6.565	< .0001	***
	ContextType	0.153	0.033	4.633	< .0001	***
	PronounType	− 0.004	0.033	− 0.112	0.91	
	ContextType:PronounType	0.064	0.066	0.966	0.33	
Region 5 *‘This’*	(Intercept)	0.087	0.035	2.532	0.018	*
	ContextType	0.097	0.028	3.442	0.001	**
	PronounType	0.019	0.027	0.709	0.48	
	ContextType:PronounType	0.097	0.06	1.624	0.11	
Region 6 *‘was’*	(Intercept)	− 0.056	0.031	− 1.804	0.077	
	ContextType	0.037	0.018	1.991	0.047	*
	PronounType	0.013	0.018	0.703	0.48	
	ContextType:PronounType	− 0.042	0.037	− 1.135	0.26	
Region 7 *Adjective*	(Intercept)	0	0.043	− 0.008	0.99	
	ContextType	0.006	0.028	0.204	0.84	
	PronounType	− 0.013	0.026	− 0.474	0.64	
	ContextType:PronounType	− 0.01	0.054	− 0.181	0.86	
Region 8 *‘But’*	(Intercept)	− 0.03	0.025	− 1.193	0.24	
	ContextType	0.007	0.021	0.346	0.73	
	PronounType	− 0.016	0.021	− 0.773	0.44	
	ContextType:PronounType	0.016	0.041	0.381	0.7	
Region 9 *‘er’/‘der’*	(Intercept)	− 0.066	0.021	− 3.052	0.005	**
	ContextType	− 0.005	0.021	− 0.251	0.8	
	PronounType	0.051	0.017	2.924	0.004	**
	ContextType:PronounType	− 0.027	0.036	− 0.741	0.46	
Region 10 *Auxiliary*	(Intercept)	− 0.101	0.017	− 6.074	< .0001	***
	ContextType	− 0.012	0.016	− 0.741	0.46	
	PronounType	− 0.006	0.016	− 0.393	0.69	
	ContextType:PronounType	− 0.029	0.032	− 0.881	0.38	
Region 11 *Adverb*	(Intercept)	− 0.192	0.017	− 11.459	< .0001	***
	ContextType	− 0.017	0.014	− 1.155	0.25	
	PronounType	0.003	0.014	0.234	0.82	
	ContextType:PronounType	− 0.021	0.029	− 0.739	0.46	

**Table 3:** Estimates for mixed effects model of residual reading times in Experiment 2 Dative Experiencer Verbs

	Fixed effects					
		Estimate	Std. Error	t value	Pr( >|t|)	
Region 1 *Dative Object / Nominative Subject*	(Intercept)	− 0.06	0.034	− 1.735	0.09	
	ContextType	− 0.041	0.022	− 1.839	0.07	
	PronounType	0.029	0.022	1.324	0.19	
	ContextType:PronounType	− 0.059	0.044	− 1.332	0.18	
Region 2 *Auxiliary*	(Intercept)	− 0.038	0.02	− 1.93	0.06	
	ContextType	− 0.023	0.018	− 1.279	0.2	
	PronounType	− 0.011	0.018	− 0.601	0.55	
	ContextType:PronounType	− 0.04	0.036	− 1.107	0.27	
Region 3 *Nominative Subject / Dative Object*	(Intercept)	− 0.119	0.029	− 4.105	< .001	***
	ContextType	0.014	0.023	0.62	0.54	
	PronounType	0.014	0.023	0.629	0.53	
	ContextType:PronounType	− 0.009	0.046	− 0.197	0.84	
Region 4 *Participle*	(Intercept)	0.4	0.067	5.965	< .0001	***
	ContextType	0.03	0.04	0.761	0.45	
	PronounType	− 0.019	0.037	− 0.514	0.61	
	ContextType:PronounType	− 0.104	0.071	− 1.456	0.15	
Region 5 *‘This’*	(Intercept)	0.14	0.036	3.856	< .001	***
	ContextType	− 0.033	0.027	− 1.234	0.22	
	PronounType	0.042	0.027	1.591	0.11	
	ContextType:PronounType	− 0.041	0.053	− 0.77	0.44	
Region 6 *‘was’*	(Intercept)	− 0.031	0.03	− 1.043	0.3	
	ContextType	− 0.039	0.021	− 1.835	0.07	
	PronounType	− 0.017	0.019	− 0.916	0.36	
	ContextType:PronounType	0.016	0.039	0.398	0.69	
Region 7 *Adjective*	(Intercept)	− 0.035	0.04	− 0.866	0.39	
	ContextType	0.021	0.027	0.785	0.43	
	PronounType	− 0.026	0.027	− 0.994	0.32	
	ContextType:PronounType	0.079	0.053	1.49	0.14	
Region 8 *‘But’*	(Intercept)	− 0.043	0.024	− 1.813	0.08	
	ContextType	− 0.009	0.018	− 0.505	0.61	
	PronounType	− 0.007	0.018	− 0.377	0.71	
	ContextType:PronounType	− 0.008	0.036	− 0.228	0.82	
Region 9 *‘er’/‘der’*	(Intercept)	− 0.046	0.024	− 1.906	0.07	
	ContextType	− 0.018	0.018	− 0.979	0.33	
	PronounType	0.036	0.021	1.719	0.09	
	ContextType:PronounType	0.029	0.036	0.817	0.42	
Region 10 *Auxiliary*	(Intercept)	− 0.096	0.019	− 5.114	< .0001	***
	ContextType	− 0.026	0.015	− 1.693	0.09	
	PronounType	− 0.046	0.017	− 2.69	0.011	*
	ContextType:PronounType	0.013	0.044	0.303	0.76	
Region 11 *Adverb*	(Intercept)	− 0.175	0.02	− 8.706	< .0001	***
	ContextType	− 0.017	0.016	− 1.024	0.31	
	PronounType	0.013	0.016	0.784	0.011	
	ContextType:PronounType	0.034	0.033	1.047	0.3	

## Footnotes

1 German has two types of demonstrative pronouns (*der* and *dieser*) that can both refer to animate entities (unlike English *this*). In the following, we focus on the processing of *der*, which is called a *d*-pronoun.

2 Note that the perfect tense was used in the context sentences, which renders the main verb in sentence-final position. This way event interpretation and thematic role assignment is completed at the sentence-final verb. With a different tense, such as present or past tense, the main verb would have been in second position and the second argument would have been the constituent at which all relevant relation become available.

3 There is an increasing body of research that investigates argument alternation in contexts and hence also considers discourse-based factors like topic, focus and givenness (e.g., Hörnig et al., 2006; Schumacher & Hung 2012; Weskott et al., 2011). But since our experimental items do not contain a rich context, we formulate our predictions on the basis of the research on argument linearization in isolated sentences.

4 Example for an active filler (i) and a passive filler (ii):

(i) Die Richterin will den Zeugen vereidigen. Das war zu erwarten. Aber er war heute abwesend.

‘The female judge wants to swear in the male witness. That was to be expected. But he was absent today.’

(ii) Der Patient wird von der Psychiaterin herein gebeten. Das war abzusehen. Aber sie wirkt sehr unaufmerksam.

‘The male patient is invited in by the female psychiatrist. That was foreseeable. But she seems very inattentive.’

5 To check whether including the effect for word length (measured in number of characters) may have artificially inflated the residual RTs for the personal pronoun *er*, which was one character shorter than the demonstrative pronoun *der*, we plotted the residual reading times for the fillers which included two types of personal pronouns, *er* and *sie,* presented after a passive context sentence. (The passive construction in the fillers was unique in the whole experiment, and hence unaffected by any potential strategies in the processing of the test items.) As can be seen in the plot in the Appendix, there are no differences in RTs between *sie* and *er* at the position of the pronoun. This suggests that residualizing does not artificially slow down the 2-character *er* vs. the 3-character *der*.

6 lmer(logRTresidual ~ Canonicity * Pronomen + (1|subject) + (1|ItemID), data = dat, REML = FALSE, control = lmerControl(optimizer = "bobyqa",optCtrl = list(maxfun = 2e5))).

7 lmer(logRTresidual ~ Canonicity * Pronomen + (1|subject) + (1|ItemID), data = dat, REML = FALSE, control = lmerControl(optimizer = "bobyqa",optCtrl = list(maxfun = 2e5))).

8 lmer(logRTresidual ~ Canonicity * Pronomen + (1 + Canonicity * Pronomen||subject) + (1 + Canonicity * Pronomen||ItemID), data = dat, REML = FALSE, control = lmerControl(optimizer = "bobyqa",optCtrl = list(maxfun = 2e5))).

9 lmer(logRTresidual ~ Canonicity * Pronomen + (1 + Canonicity * Pronomen||subject) + (1|ItemID), data = dat, REML = FALSE, control = lmerControl(optimizer = "bobyqa",optCtrl = list(maxfun = 2e5))).

10 lmer(logRTresidual ~ Canonicity * Pronomen + (1 + Canonicity * Pronomen||subject) + (1|ItemID), data = dat, REML = FALSE, control = lmerControl(optimizer = "bobyqa",optCtrl = list(maxfun = 2e5))).

11 lmer(logRTresidual ~ Canonicity * Pronomen + (1 + Canonicity * Pronomen|subject) + (1 + Canonicity * Pronomen|ItemID), data = dat, REML = FALSE, control = lmerControl(optimizer = "bobyqa",optCtrl = list(maxfun = 2e5))).

12 lmer(logRTresidual ~ Canonicity * Pronomen + (1 + Canonicity * Pronomen|subject) + (1 + Canonicity * Pronomen|ItemID), data = dat, REML = FALSE, control = lmerControl(optimizer = "bobyqa",optCtrl = list(maxfun = 2e5)));
